# Development and Characterisation of a New In Vitro Murine Mucosal Mast Cell Model

**DOI:** 10.1111/all.70022

**Published:** 2025-08-29

**Authors:** Louise Battut, Jasper Kamphuis, Nadine Serhan, Laurent Reber, Nicolas Cenac, Gilles Dietrich, Eric Espinosa

**Affiliations:** ^1^ Institut de Recherche en Santé Digestive (IRSD), INSERM U1220, INRA, INP‐ENVT, University of Toulouse Toulouse France; ^2^ Toulouse Institute for Infectious and Inflammatory Diseases (Infinity), UMR 1291, INSERM, CNRS, University of Toulouse Toulouse France

**Keywords:** allergy, mast cell, mucosal mast cell model

To the Editor,

Mouse mast cells (MCs) fall into two subpopulations with well‐defined roles and characteristics: connective tissue mast cells (CTMCs) and mucosal mast cells (MMCs) [1, 2]. While in vitro models of CTMCs exist in humans and mice, a reliable and relevant model of MMCs is still lacking. While a few previously described protocols have used the addition of TGF‐β1 and IL‐9 alongside IL‐3 and SCF from the onset of bone marrow cell culture to generate MMC‐like cells, these approaches often vary in culture duration and in the nature of the resulting cells [3, 4]. Here, we propose a two‐step protocol that more faithfully reflects the two major stages of MMC differentiation, enabling the generation of mouse bone marrow‐derived mucosal mast cells (BM‐MMCs) in vitro. Bone marrow cells were cultured in a complete Opti‐MEM medium supplemented with IL‐3 and SCF for 4 weeks to induce MC commitment before adding for one additional week both IL‐9 and TGF‐β1 to promote MMC proliferation and maturation [2, 3, 5] (Figures 1A and S1). This delayed addition of IL‐9 and TGF‐β1 resulted in a higher percentage of cells showing an MMC phenotype compared with its addition at the start of culture (Figure S2). BM‐MMCs were phenotypically and functionally compared to the previously established CTMC model (PCMCs) [6]. In 5 weeks, this two‐step differentiation protocol produces approximately 35 million MCs (36 ± 9 million, *n* = 7 mice) from 1 million bone marrow cells (Figure S3A). After 7 weeks in culture, the BM‐MMC showed more than 95% viability (Figure S3B). BM‐MMCs expressed the MC markers FcεRI, CD117 and ST2 (IL‐33 receptor) together with the typical MMC markers CD103 and MCPT1 (Figure 1B–D) and showed IL‐3 dependency (Figure S4). Furthermore, these cells did not stain positively for avidin (which binds to heparin contained in the granules of CTMCs) (Figure 1D) and exhibited a reduced granular mass, histamine, and MCPT6 contents as compared to their PCMC counterparts (Figure 1E–G). BM‐MMCs degranulated in response to FcεRI aggregation but not to the 48/80 compound secretagogue, as expected for MMCs which do not express its receptor Mrgprb2 (Figure 1H–J).

**FIGURE 1:**
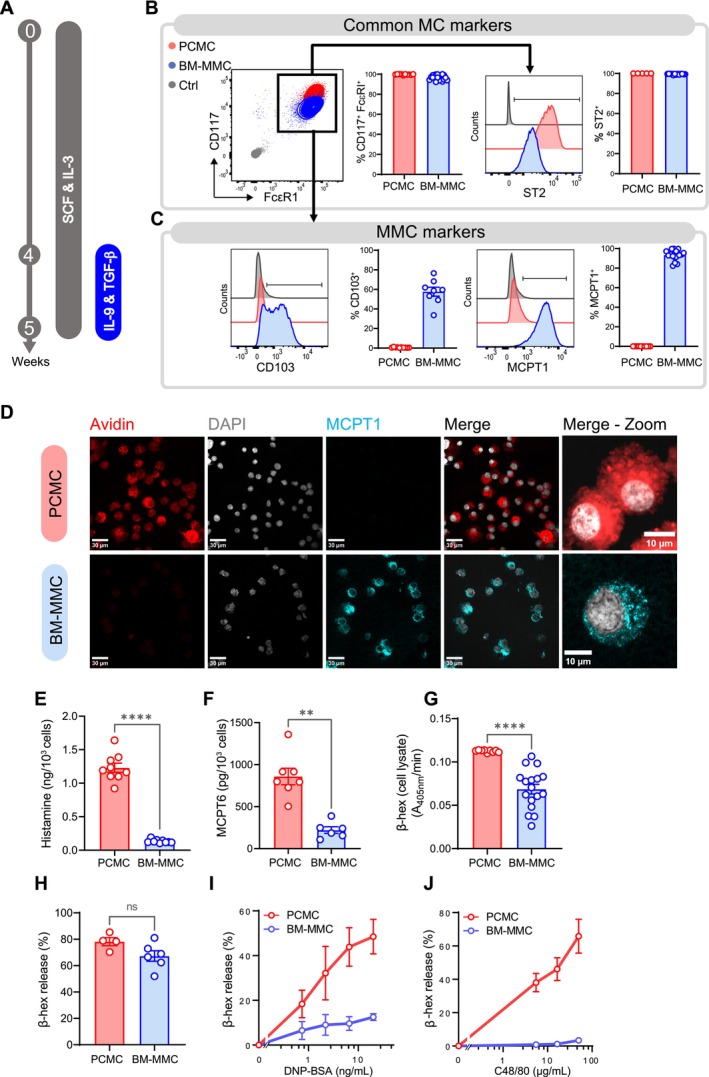
BM‐MMCs exhibit phenotypic and functional features of MMCs. (A) Experimental procedure: Bone marrow cells were cultured with SCF and IL‐3 for 4 weeks before being supplemented with IL‐9 and TGF‐β1 for 1 week. (B‐C) Flow cytometry analysis of BM‐MMCs and PCMCs: Representative dot plot and histogram overlays of PCMCs (red), BM‐MMCs (blue) and unstained control (grey) gated on single live cells; and pooled data from *n* = 5 independent experiments. (D) Representative confocal microscopy images of avidin sulforhodamine (red), MCPT1 protease (cyan) and DAPI (grey) staining. (E, F) Cellular content in histamine (E) and in MCPT6 protease (F). (G) Granular mass evaluated by measuring total β‐hexosaminidase content after cell lysis. (H–J) BM‐MMC and PCMC degranulation in response to PMA/ionomycin (H), DNP‐BSA challenge (on anti‐DNP IgE‐sensitised MCs) (I) or C48/80 (J). Degranulation was measured by the standard β‐hexosaminidase release assay. Pooled data from 3 (E, F), 4 (G, I) or 2 (H, J) independent experiments, Each data point represents an individual mouse. Mann–Whitney test, ****p < 0.0001, **p < 0.01, *p < 0.05.

Transcriptomic analysis showed clear differences in gene expression between the two cell types. Among the 40 most variable genes, the typical MMC genes *Mcpt1*, *Mcpt2, Mcpt8 and Itgae*, and the typical CTMC genes *Mrgprb2* and *Mrgprb1* were clearly clustered in BM‐MMCs and PCMCs, respectively (Figure S5A). We found 5174 differentially expressed genes (DEGs, Padj < 0.01 and Fold change > 2) (Figure 2A and Table S1) between BM‐MMCs and PCMCs. Further analysis of the expression of the prototypical MC genes substantiated that BM‐MMCs displayed MMC features, including a diminished ability to produce histamine and the expression of genes involved in chondroitin sulfate synthesis (Figure 2B–D) (5). This analysis also revealed that receptors relevant to MC biology were differentially regulated between BM‐MMC and PCMC (Figure 2E).

**FIGURE 2:**
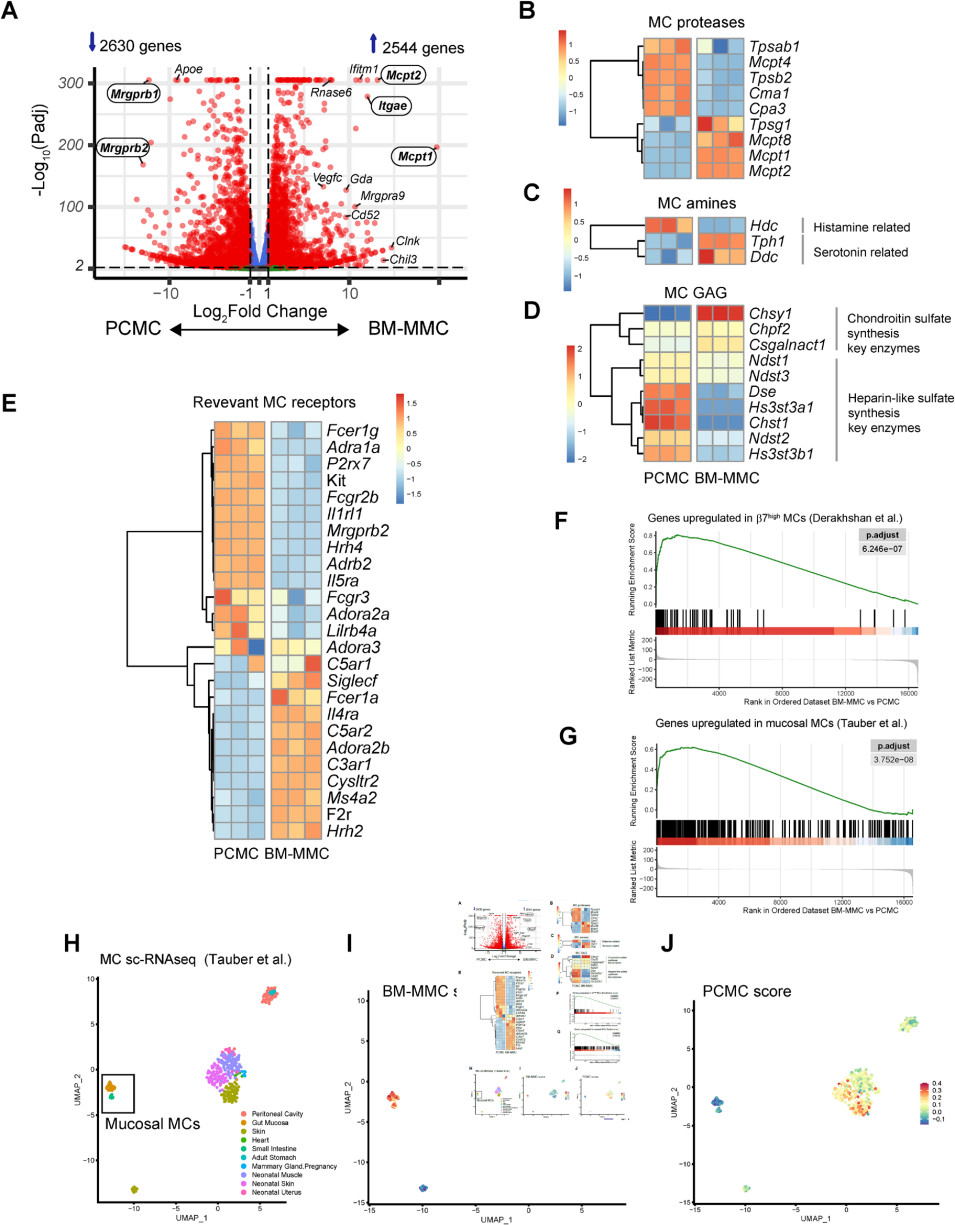
BM‐MMC transcriptome shows typical MMC characteristics. (A) Volcano plot of DEGs between BM‐MMCs and PCMCs. Typical MMC and CTMC genes are highlighted. (B–D) Heatmaps showing the expression of MC genes related to granule protease content (B), biogenic amine biosynthesis (C), glycosaminoglycan biosynthesis (D) and the expression of relevant MC receptors (E). (F–G) Gene set enrichment analysis plots from integrin β7^high^ lung MMC genes (black bars) described by Derakhshan et al. (F) and from intestinal MMCs identified by Tauber et al. (G) found in our dataset ranked genes (descending Log_2_FC). Green line shows the enrichment score for each gene. (H–J) MC single cell RNA‐Seq data from different organs were integrated by Tauber et al. (H). This dataset was used to compute an enrichment score of the 100 most upregulated genes found in our BM‐MMCs (I) or PCMCs (J).

We next investigated whether the genetic signature of BM‐MMCs could be found in MMCs described in the literature. Based on a transcriptional analysis of β7 integrin^High^ (MMCs) versus β7 integrin^Low^ (CTMCs) lung MCs from HDM‐challenged mice [2], gene set enrichment analysis (GSEA) indicated that genes overexpressed in β7^High^ MCs were significantly enriched in BM‐MMCs (Figure 2F). Likewise, GSEA analysis of sc‐RNA‐Seq dataset from mouse MCs isolated from different tissues, which exhibited a clear clustering of MMCs (Mrgprb2^−^) and CTMCs (Mrgprb2^+^) [1] showed that the MMC gene signature was significantly enriched in BM‐MMCs (Figure 2G). Reciprocally, the enrichment score of the 100 upregulated genes in BM‐MMCs revealed a transcriptomic signature similar to that of mouse MMCs identified by Tauber et al. [1] (Figure 2H–J). Moreover, the RNA‐Seq analysis of the genes coding pattern recognition receptors and antimicrobial molecules underlined the distinct functional roles of BM‐MMCs and PCMCs and the obvious antimicrobial capacity of BM‐MMCs (Figure S5B,C).

Thus, BM‐MMCs, which have transcriptomic characteristics similar to MMCs, can be an effective tool for studying a significant number of viable MMCs during a 15‐day period in vitro.

## Author Contributions

Experimental design: E.E., L.B.; Conducting experiments: L.B., N.S.; Statistical analysis: E.E., L.B.; Writing (original draft): E.E., L.B., G.D.; Writing (review and editing): all authors.

## Conflicts of Interest

The authors declare no conflicts of interest.

## Supporting information


**Figure S1:** Determining the optimal timing for adding IL‐9 and TGF‐β1. Bone marrow cells were cultured in complete Opti‐MEM medium with SCF and IL‐3, and supplemented with IL‐9 plus TGF‐β1 either from Day 1 of culture or after 1, 2, 3 or 4 weeks. After 1 week in the IL‐9 plus TGF‐β1‐supplemented medium, surface expression of CD117 and FcεR1 and intracellular expression of MCPT‐1 were analysed by flow cytometry. (A) Diagram of experimental protocol. (B) Representative dotplots and histograms. (C) Pooled data from five independent experiments.
**Figure S2:** Addition of IL‐9 and TGF‐β1 at the start of culture results in low MMC phenotype induction. Bone marrow cells were cultured in complete Opti‐MEM medium supplemented with IL‐3, SCF, IL‐9 and TGF‐β1 for a period of 3–7 weeks. Surface expression of CD117 and FcεR1 and intracellular expression of MCPT1 were analysed by flow cytometry. (A) Diagram of experimental protocol. (B) Representative dotplots and histograms. (C) Pooled data from three independent experiments.
**Figure S3:** BM‐MMC culture yield and cell viability. (A) BM‐MMC number obtained per 10^6^ seeded cells (pooled data from two independent experiments, *n* = 6 mice). (B) Flow cytometry analysis of BM‐MMC viability after seven weeks in culture, representative experiment and pooled data from 3 independent experiments, each data point represents an individual mouse, median ± 95% confidence interval.
**Figure S4:** BM‐MMCs show IL‐3 dependency. After five weeks in culture (Figure 1A), IL‐3 concentration was reduced and cell viability was measured seven days later.
**Figure S5:** RNA‐Seq analysis of BM‐MMCs compared with PCMCs. (A) Heatmap of the 40 most variable genes (with the highest variance across all samples), (B, C) heatmaps of genes encoding antimicrobial molecules (B) and pattern recognition receptors (C); gene lists were retrieved from the UniprotKB database and manually curated. Only genes with TPM (transcript per million) > 1 were shown. The Heatmaps clustered genes according to their relative expression. Log_2_FoldChange and FDR (BM‐MMCs vs. PCMCs), and TPM were also plotted.

**Table S1:** Differentially expressed genes (|Log_2_FoldChange| > 1 and FDR < 0.01) between BM‐MMCs and PCMCs. Summary of statistical analysis with DESeq2 package and gene annotations.

## Data Availability

The data that support the findings of this study are openly available in Gene Expression Ombibus at https://www.ncbi.nlm.nih.gov/geo/query/acc.cgi, reference number GSE295774.
