# Supplementary material for: Development and Characterisation of a New In Vitro Murine Mucosal Mast Cell Model
Source: Allergy. 2025 Aug 29;80(10):2926–30. doi: 10.1111/all.70022 (PMC12486343; doi:10.1111/all.70022)
Supplement: Supplementary file 1 — Figure S1: Determining the optimal timing for adding IL‐9 and TGF‐β1. Bone marrow cells were cultured in complete Opti‐MEM medium with SCF and IL‐3, and supplemented with IL‐9 plus TGF‐β1 either from Day 1 of culture or after 1, 2, 3 or 4 weeks. After 1 week in the IL‐9 plus TGF‐β1‐supplemented medium, surface expression of CD117 and FcεR1 and intracellular expression of MCPT‐1 were analysed by flow cytometry. (A) Diagram of experimental protocol. (B) Representative dotplots and histograms. (C) Pooled data from five independent experiments. Figure S2: Addition of IL‐9 and TGF‐β1 at the start of culture results in low MMC phenotype induction. Bone marrow cells were cultured in complete Opti‐MEM medium supplemented with IL‐3, SCF, IL‐9 and TGF‐β1 for a period of 3–7 weeks. Surface expression of CD117 and FcεR1 and intracellular expression of MCPT1 were analysed by flow cytometry. (A) Diagram of experimental protocol. (B) Representative dotplots and histograms. (C) Pooled data from three independent experiments. Figure S3: BM‐MMC culture yield and cell viability. (A) BM‐MMC number obtained per 106 seeded cells (pooled data from two independent experiments, n = 6 mice). (B) Flow cytometry analysis of BM‐MMC viability after seven weeks in culture, representative experiment and pooled data from 3 independent experiments, each data point represents an individual mouse, median ± 95% confidence interval. Figure S4: BM‐MMCs show IL‐3 dependency. After five weeks in culture (Figure 1A), IL‐3 concentration was reduced and cell viability was measured seven days later. Figure S5: RNA‐Seq analysis of BM‐MMCs compared with PCMCs. (A) Heatmap of the 40 most variable genes (with the highest variance across all samples), (B, C) heatmaps of genes encoding antimicrobial molecules (B) and pattern recognition receptors (C); gene lists were retrieved from the UniprotKB database and manually curated. Only genes with TPM (transcript per million) > 1 were shown. The Heatmaps clustered genes a [file ALL-80-2926-s002.docx]

**Supplementary Materials**

**Supplementary methods**

- **Cell culture**

PCMCs: Peritoneal cells from C57BL/6 mice (aged 7 to 14 weeks old) were collected by peritoneal lavage with 5 mL of PBS + 2 mM EDTA. Cells were filtered through a 70 µm strainer, washed and seeded in a 24-well culture plate at 10^6^ cells/mL in Opti-MEM Glutamine supplemented with 10% FBS, 100 IU/mL penicillin, 100 μg/mL streptomycin, 50 µM 2-Mercaptoethanol, MEM Non-Essential Amino Acids Solution (all provided from Gibco), 3% supernatant of Chinese hamster ovary transfectants secreting murine stem cell factor (a gift from Dr P. Dubreuil, Inserm U891). Twenty-four hours later, nonadherent cells were harvested in a new well. Three days later, nonadherent cells and adherent cells harvested by flushing were pelleted and resuspended in fresh culture medium at a concentration of 3 to 5x10^5^ cells/mL. The same procedure was repeated twice a week. PCMCs were characterized by flow cytometry (CD117^+^, FcεR1^+^, ST2^+^, MCPT-1^-^), confocal microscopy (MCPT-1^-^, avidin^+^) and functional degranulation assays.

BM-MMCs: Bone marrow cells of 7-14 weeks old C57BL/6 mice were washed out of the tibia and femur and were passed through a 70 µm strainer, washed and seeded (about 30 million cells) in a T75 flask in Opti-MEM Glutamine supplemented with 10% FBS, 100 IU/mL penicillin, 100 μg/mL streptomycin, 50 µM 2-Mercaptoethanol, MEM Non-Essential Amino Acids Solution (all from Gibco), 3% supernatant of Chinese hamster ovary transfectants secreting murine stem cell factor (a gift from Dr P. Dubreuil, Inserm U891) and 5 ng/mL of recombinant murine IL-3 (Peprotech, ThermoFisher Scientific). Cells were diluted by half in fresh medium every 2 days to maintain a concentration of 5x10^5^ to 1x10^6^ cells/mL and the flasks were changed every 5 days to remove adherent cells. Cells were maintained in this culture medium for 4 weeks (unless otherwise indicated in the figures) and then supplemented with 5 ng/mL recombinant murine IL-9 (Peprotech, ThermoFisher Scientific) and 1 ng/mL of recombinant murine TGF-β1 (Biolegend). The medium was renewed every 3 days and the cells maintained at a concentration of 5x10^5^ to 1x10^6^ cells/mL. After one week, cells were characterized by flow cytometry for CD117, FcεR1, ST2 and MCPT1 expression and by confocal microscopy (MCPT1, avidin) and degranulation assays.

- **Flow cytometry**

For cell surface molecules staining, cells were incubated with fixable viability dye (eFluor780 eBioscience) and fluorochrome-labeled primary antibodies in PBS, 10% FBS, 2 mM EDTA (FACS Buffer) at 4°C for 30 minutes (CD117-PEVio770 1:100 and FcεR1-APC 1:100 Recombinant Engineered Antibody from Miltenyi, ST2-PE 1:100 clone U29-93 from BD Bioscience and CD103-BV650 1:150 clone 2E7 from Biolegend). Cells were then washed and fixed in 4% paraformaldehyde for 15 minutes at RT. For intracellular MCPT1 staining, cells were permeabilized in FACS buffer with 0,1% saponin (permeabilization buffer) and then incubated with rat IgG1 anti mouse MCPT-1 primary antibody or isotype matched control (eBioscience) for 2 hours at RT, next washed and incubated with Goat anti rat IgG Secondary Antibody, Alexa Fluor™ 488 (Invitrogen) for 1 hour at RT. Cells were washed and resuspended in FACS Buffer and data were acquired on a Fortessa X20 (BD Biosciences) and further analyzed using the FlowJo software (Tree Star, Ashland, OR, USA). Debris, doublet cells and dead cells were excluded based on FSC/SSC analysis and viability dye staining.

- **Confocal microscopy**

Cells were transferred onto poly-L-lysine (Sigma-Aldrich) –coated slides at a concentration of 3x10^5^ cells/mL and then fixed with 4% paraformaldehyde during 20 minutes. Cells were permeabilized and blocked in PBS, 5% SVF, 1%BSA, 0.1% saponin and stained with rat IgG1 anti mouse MCPT1 primary antibody (eBioscience) for 2 hours at RT. After 3 washes, cells were stained with Goat anti rat IgG Secondary Antibody, Alexa Fluor™ 488 (Invitrogen) plus 2 μg/mL avidin-sulforhodamine 101 (Sigma-Aldrich) for 1 hour at RT and counterstained with DAPI (1 µg/mL, Invitrogen). The samples were mounted in Fluoroshield™ mounting medium and examined with a Zeiss LSM 710 confocal microscope.

- **Degranulation assay**

Cells were sensitized with anti-dinitrophenyl (DNP) IgE (clone SPE-7, Sigma-Aldrich) for 16 hours in culture medium. Cells were next washed and distributed in 96-well U-bottom plates at a density of 1x10^5^ cells in 50 µL Tyrode’s buffer 0.1% BSA and adapted to 37°C for 40 minutes. Cells were then stimulated with indicated concentrations of DNP-BSA or C48/80 or 100 ng/mL PMA + 1µg/mL ionomycin for 30 min at 37°C, 5% CO_2_. To measure total β-hexosaminidase content, cells were lysed with 0,5% triton X-100 (100,000 cells/100 µL). 20 µL of the supernatants were mixed with 50 µL of 1.3 mg/mL of p-nitrophenyl N-acetyl-β-D-glucosaminide in citrate buffer (0.1 mol/L, pH 4.5) for 30 min. Reaction was stopped with 150 µL glycine buffer (0.2 mol/L, pH 10.7) and absorbance at 405 nm was measured on an EnSight™ Plate Reader (PerkinElmer).

- **ELISA**

Histamine quantitation was performed using the Histamine Competitive ELISA Kit (invitrogen), following the manufacturer’s instructions. Briefly, samples or standards were incubated in the histamine pre-coated wells in the presence of the biotinylated detection antibody for 45 minutes at 37°C. After washing, HRP Conjugate Solution was added into each well for 30 min at 37°C. After washing, the substrate was incubated for 10 minutes before stopping the reaction and measuring the absorbance at 450 nm.

MCPT6 concentrations were measured using the Mouse Mast Cell Protease 6 ELISA Kit (invitrogen), according to the manufacturer's protocol. Standards and samples were added in pre-coated microplate wells with a monoclonal antibody anti-MCPT6. After 2.5 hours of incubation at room temperature, wells were washed and incubated with biotinylated anti-MCPT6 antibody for 1 hour. Following additional washes, streptavidin-HRP conjugate were added for 45 minutes. Plates were washed again, and TMB substrate was added to each well. The colorimetric reaction was stopped after 30 min using stop solution.

- **Bulk RNA sequencing**

Cells from PCMC lines (n=3) and from BM-MMC lines (n=3) (tested phenotypically and functionally as CTMCs and MMCs) were harvested and total RNA was extracted using TRIzol (Invitrogen) and purified on a RNeasy column (RNeasy Mini Kit Qiagen 79254). Libraries were prepared using 418-500 ng of high-quality total RNA. Illumina Stranded Total RNA Prep Ligation with Ribo-Zero Plus kit (Illumina) and IDT for Illumina DNA/RNA UD Indexes (Illumina) are used according to the manufacturer protocol. Size selection was performed using SPRIselect beads (Beckman Coulter). Library size and quality were confirmed on HS NGS Fragment kit for Fragment Analyzer (Agilent). Libraries were quantified by qPCR using the KAPA Library quantification kit for Illumina platforms (Roche Sequencing Solution). Samples were pooled in equimolar fashion. The libraries were sequenced on a Novaseq 6000 (Illumina) platform in paired end sequencing 2 × 150 bp with 10bp-dual indexes. Fastq files were generated via llumina bcl2fastq2.

- **RNA-seq analysis**

Quality check of raw reads was performed with FastQC (v0.11.9) and transcript abundance was quantified using Salmon (v1.9.0) via Galaxy interface (1), based on the transcript fasta files downloaded from Ensembl.org (Mus_musculus.GRCm39.cdna.all.fa). Salmon was run with the default settings using GC bias correction. Transcript abundances were aggregated to gene-level abundances using tximport function (tximport package v1.32.0) and differential expression analysis was carried out by DESeq2 package (v1.44.0) in R (v4.4.1). Transcripts were considered differentially regulated between the two MC types (*n* = 3) if |log_2_ fold-change | ≥ 1 and adjusted *P* value ≤ 0.01. Volcano plots were generated using Enhanced volcano package (v1.22.0). Heatmaps were generated using pheatmap package (v1.0.12) and rows were clustered with Ward.D2 method or ComplexHeatmap package (v2.20.0) for PRRs and AMPs.

Gene set enrichment analysis (GSEA) was performed on pre-ranked gene list (according to Log2FoldChange) using GSEA (2) function of clusterProfiler package (v4.12.6) with two custom gene sets: 73 lung MMC genes were from Derakhshan et al. dataset, i.e. genes upregulated in lung MMC (β7^high^) vs CTMC (β7^low^) upon HDM treatment, retrieved from table S1 (FoldChange>2 and FDR<0.05 and not ribosomal protein coding) and 275 genes were retrieved from table S2, Tauber et al. identified as upregulated in MMCs from different organ as compared to CTMCs. Enrichment plots for GSEA were produced using the gseaplot2 function in the enrichplot package (v1.24.4). Mice MC single cell RNAseq data integrated by Tauber et al. were used to compute an enrichment score using AddmoduleScore function (package Seurat v4.1.3) based on our 100 most upregulated genes found in our BM-MMC or PCMC lines.

**Supplementary figures**

**
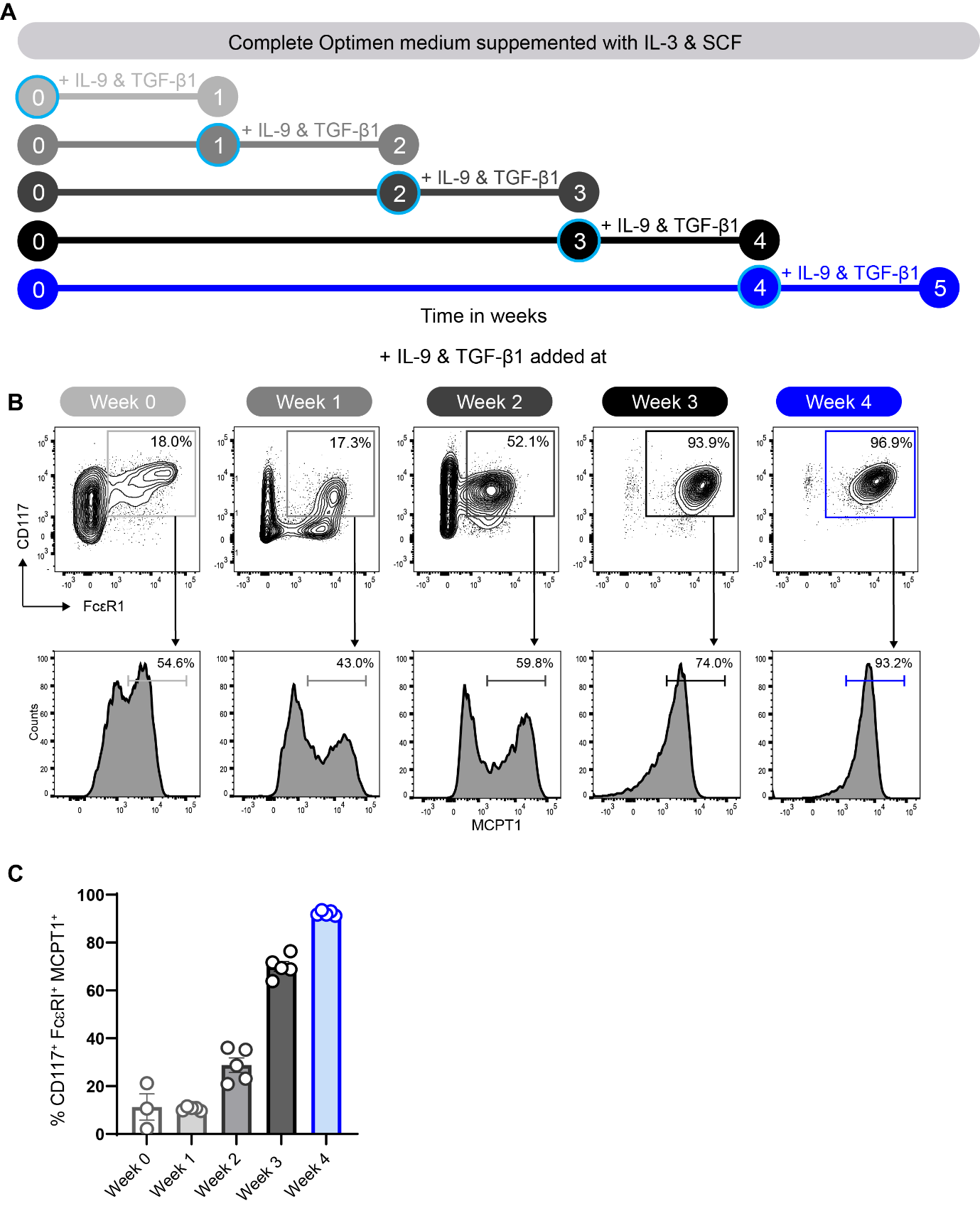
**

**Figure S1. Determining the optimal timing for adding IL-9 & TGF-β1.**  Bone marrow cells were cultured in complete Opti-MEM medium with SCF and IL-3, and supplemented with IL-9 plus TGF-β1 either from day 1 of culture or after 1, 2, 3 or 4 weeks. After one week in the IL-9 plus TGF-β1-supplemented medium, surface expression of CD117 and FcεR1 and intracellular expression of MCPT-1 were analysed by flow cytometry (A) Diagram of experimental protocol. (B) Representative dotplots and histograms. (C) Pooled data from 5 independent experiments.


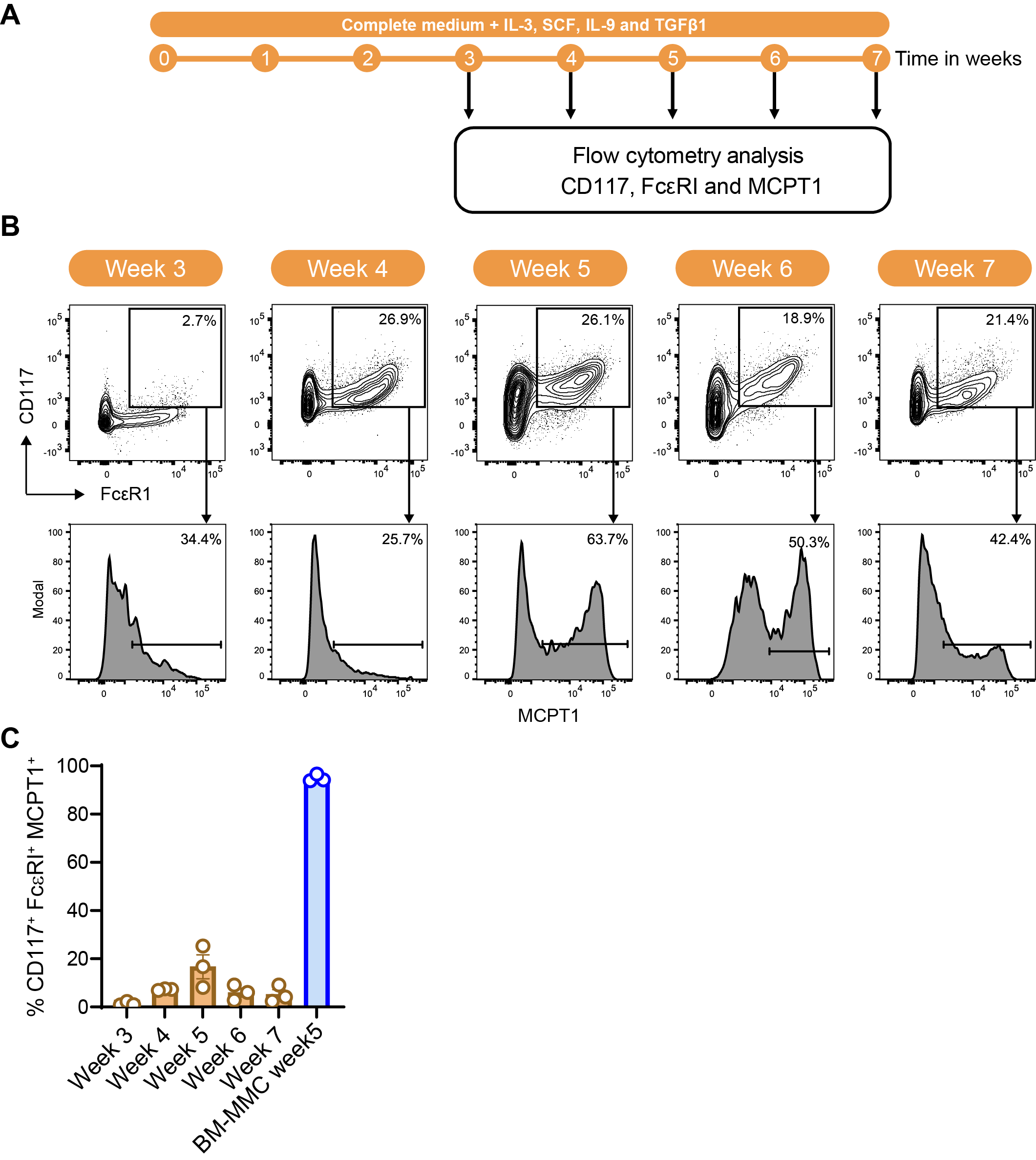


**Figure S2. Addition of IL-9 & TGF-β1 at the start of culture results in low MMC phenotype induction.**  Bone marrow cells were cultured in complete Opti-MEM medium supplemented with IL-3, SCF, IL-9 and TGF-β1 for a period of 3 to 7 weeks. Surface expression of CD117 and FcεR1 and intracellular expression of MCPT1 were analysed by flow cytometry (A) Diagram of experimental protocol. (B) Representative dotplots and histograms. (C) Pooled data from 3 independent experiments.


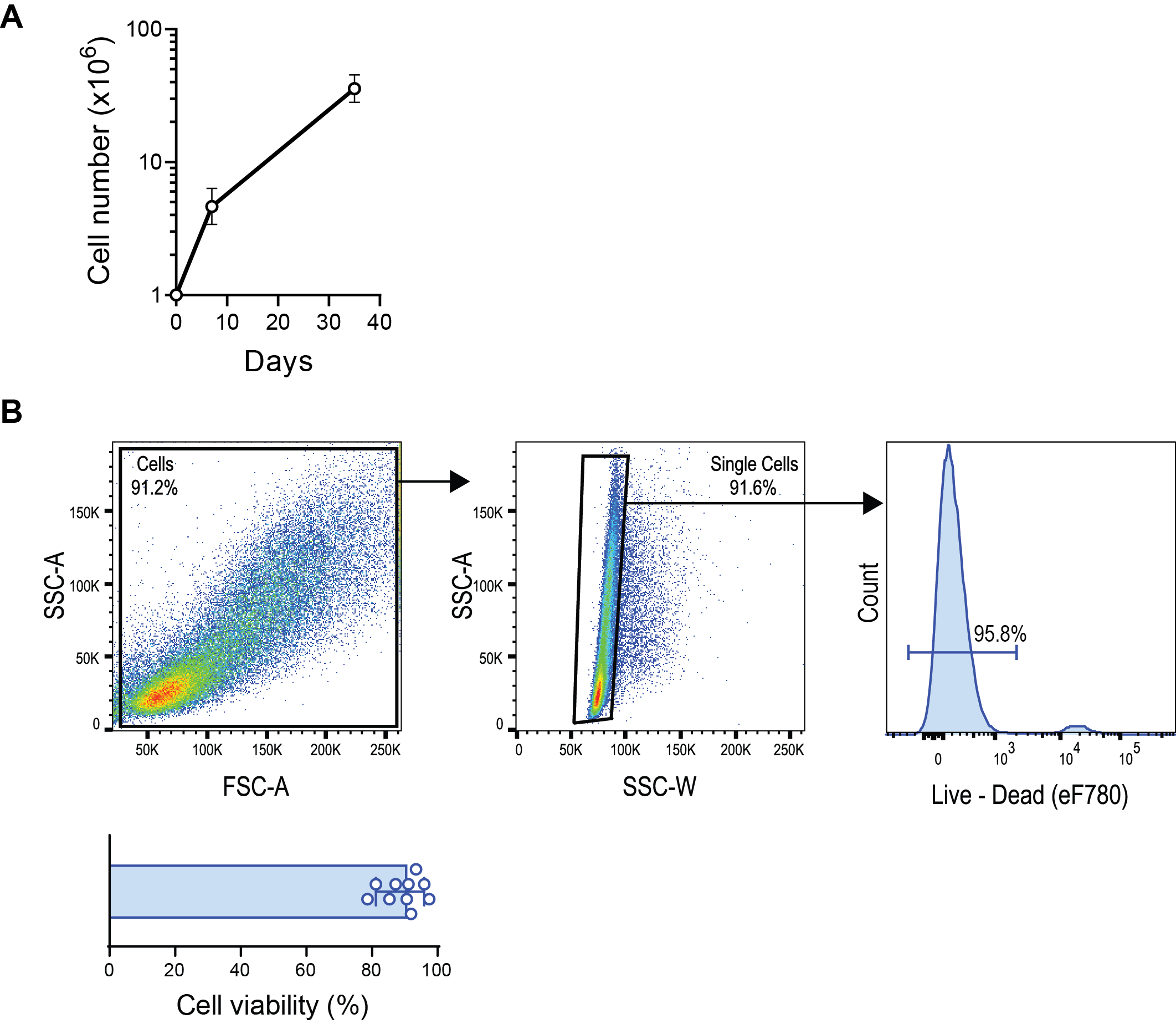


**Figure S3. BM-MMC culture yield and cell viability.**

(A) BM-MMC number obtained per 10^6^ seeded cells (pooled data from two independent experiments, n=6 mice) (B) Flow cytometry analysis of BM-MMC viability after 7 weeks in culture, representative experiment and pooled data from 3 independent experiments, each data point represents an individual mouse, median ± 95% confidence interval

**
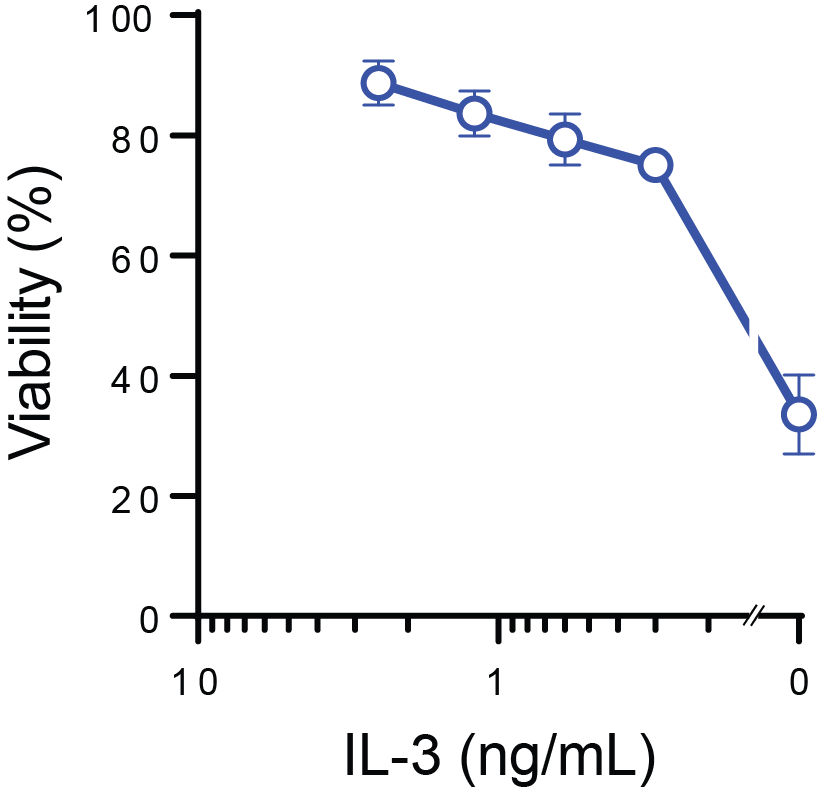
**

**Figure S4. BM-MMCs show IL-3 dependency.**

After 5 weeks in culture (Figure 1A), IL-3 concentration was reduced and cell viability was measured 7 days later.

**
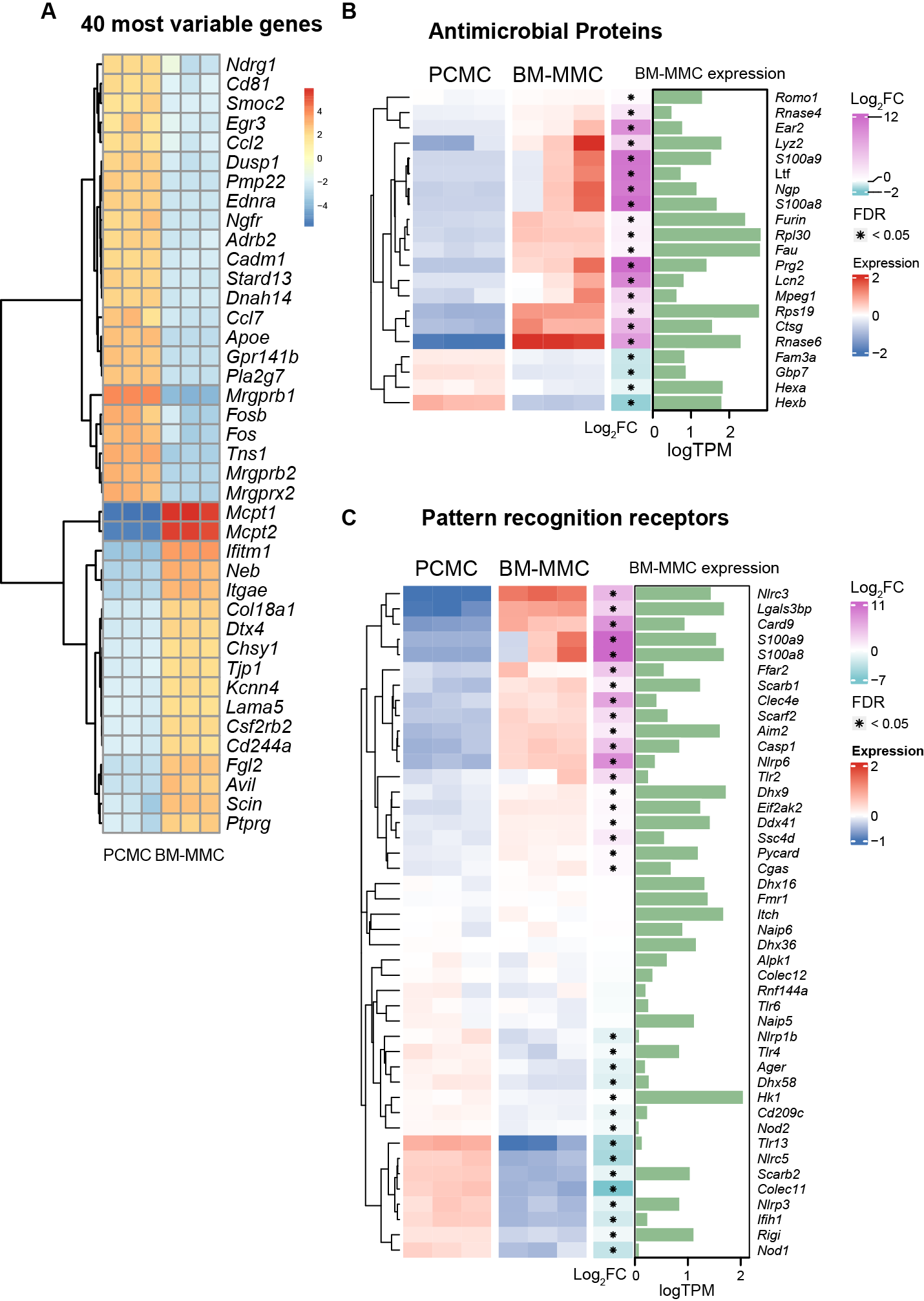
**

**Figure S5. RNA-seq analysis of BM-MMCs compared with PCMCs.** (A) Heatmap of the 40 most variable genes (with the highest variance across all samples), (B-C) Heatmaps of genes encoding antimicrobial molecules (B) and pattern recognition receptors (C); gene lists were retrieved from the UniprotKB database and manually curated. Only genes with TPM (transcript per million)>1 were shown. The Heatmaps clustered genes according to their relative expression. Log_2_FoldChange and FDR (BM-MMCs vs. PCMCs), and TPM were also plotted.

**Supplementary table**

**Table S1. Differentially Expressed Genes (|Log_2_FoldChange|>1 and FDR<0.01) between BM-MMCs and PCMCs**. Summary of statistical analysis with DESeq2 package and gene annotations.
